# Assessment of different types of intra oral scanners and 3D printers on the accuracy of printed models: An *in vitro* study

**DOI:** 10.4317/jced.58765

**Published:** 2021-12-01

**Authors:** Fernando Igai, Washington-Steagall Junior, Carolina-Mayumi Iegami, Pedro-Tortamano Neto

**Affiliations:** 1 Universidade de São Paulo, Department of Prosthodontics, School of Dentistry; São Paulo, SP, Brazil; 2 Universidade Nove de Julho, School of Dentistry; São Paulo, SP, Brazil

## Abstract

**Background:**

3D printing technology is a reality in Dentistry and presents several ways to obtain a printed model. The aim of this study was to verify the influence of different types of intraoral scanners and 3D printers on the accuracy of printed models in comparison to plaster models obtained from conventional impressions.

**Material and Methods:**

A dental study model was used as the reference model and was molded with polyvinyl siloxane to produce the plaster models. It was also scanned with two types of intraoral scanners and the digital files were printed by two types of 3D printers. The plaster and printed models formed five groups (n=50), which were analyzed using linear measurements at six dimension sites. In order to test the equivalence in the precision of the measurements made in the reference model and in the different models of the experimental groups, the Schuirmann Two-One Sided t-test was applied. The trueness of the measurements of the experimental models was tested in comparison to those of the reference model by applying tests for paired data. In all statistical tests, the significance level of 5% (α = 0.05) was adopted.

**Results:**

In relation to precision, all five groups presented similar and acceptable results. The trueness analysis indicated that both the printed and the plaster models had average measurements that were different from the reference model.

**Conclusions:**

It was concluded that the accuracy of printed and plaster models was impaired due to the trueness of the models. The type of printer influenced the accuracy of the printed models, while the type of scanner did not. The standardization of the method of obtaining printed models must be carried out in order to provide the production of quality models. However, there will be differences between the technologies.

** Key words:**Dental models, three-dimensional printing, dimensional accuracy.

## Introduction

The use of printed models is a reality in Dentistry. 3D printers produce models that enable laboratory (1,2) and clinical (3) applications, providing an efficient workflow. There are several ways to obtain printed models, such as stereolithography, digital light processing and polyjet printers, among others. The printed models can be made with different types of materials (4). The main characteristic of the models obtained by 3D printing is their manufacture through the deposition of the material in an additive and selective manner, which, unlike milling, builds the physical model without wearing and tearing, but through the addition of material. This process saves material and allows models with varied geometric shapes (5).

Several steps must be analyzed when obtaining printed models, as they influence the overall quality of the model: the scanner that generates the digital file (6,7), the scanning strategy (8-10), operator experience (11) and the type of printer (12-14) are some of them. All of these variables impact the accuracy of the models, since they can add error to the process if not executed the proper way.

Accuracy consists of the conjunction of two terms, precision and trueness. The term precision refers to the ability of a method to generate several models with similar measures. In this context, an imprecise method would result in the production of different sized models, which is not desirable, since a single reference model is being replicated. On the other hand, trueness refers to the ability of a method to generate models with measures that are similar to the reference model to be replicated. The final desired feature for a model-generating method is to present clinically accepTable precision and trueness (15-18,14).

 The method of comparative analysis of the models used in Dentistry, in many cases, is based on scanning protocols of the physical models with a reference scanner and, after this process, the analysis is carried out using image superimposing programs (19-21,10,14). These programs overlap the models of the experimental groups in relation to the reference model, thus demonstrating which experimental group has the best accuracy. However, in these types of analysis, the texture and surface finish of the physical model are not taken into consideration, since the model is virtual. Another method of analysis with computer programs is the linear measurement (22-24,7,18) that performs the comparison between the models by means of linear measurements.

Plaster models, obtained through conventional impressions, are still widely used and have established clinical applicability, within their characteristics. However, the plaster model may have problems related to the quality of the impression or its manufacture, which can compromise the quality of the work to be performed (25). The physical and virtual models that are used in oral rehabilitation demand a clinically acceptable accuracy (26). Therefore, it is necessary that the printed models and plaster models are analyzed to verify their accuracy in relation to a reference model, taking into account the influence of surface characteristics and their general finish.

The aim of this study was to verify the influence of different types of intra oral scanners and 3D printers on the accuracy of printed models in comparison to plaster models obtained from conventional impressions. The null hypothesis is that there are no significant differences in the accuracy of the printed and plaster models used in the present study, in comparison to the reference model.

## Material and Methods

A dental study model (P-Oclusal, São Paulo, Brazil) served as reference for the making of the experimental models. It featured intact dental elements and elements with partial and total dental preparations for prosthetic purposes.

The printed experimental models were obtained from scans made by two different types of intraoral scanners, and were printed on two 3D printers. The Trios Pod Colors intraoral scanner (3shape, Copenhagen, Denmark) and the Cerec Omnicam intraoral scanner (Sirona, Bensheim, Germany) were used by operators who were trained by the manufacturers.

A single scanning strategy, recommended by the scanners manufacturers, was adopted for both scanners. The scanning started on the occlusal surface of the Maxillary right second molar of the reference model, followed by the occlusal surfaces of the upper teeth until the occlusal surface of the Maxillary left second molar was reached. The scanning continued through the buccal surfaces of the teeth, starting with the buccal surface of the Maxillary left second molar until the buccal surface of the Maxillary right second molar was reached. Soon afterwards, the palatal surfaces of the teeth were scanned, starting with the palatal surface of the Maxillary right second molar, continuing with the scanning until reaching the palatal surface of the Maxillary left second molar.

After obtaining the scan files of the reference model, the models were printed. Miicraft 125 series (Miicraft, Amityville, USA) and Eden 500V (Stratasys, Eden Prairie, USA) printers were used. The Miicraft 125 series printer performs printing using the DLP method. The layer thickness that was used in the study was of 5 µm. The printing of a set of two models in the horizontal position took approximately two hours. After this process, the model was transferred to the Visio Beta Vario® model polymerizer (3M, Two Harbors, USA) which carried out the final polymerization of the model. Each set of two models was kept in the polymerizer for 14 minutes. Ten models were printed from the Trios intraoral scanner and ten models from the Omnicam intraoral scanner, totaling twenty models printed by the Miicraft 125 series printer.

The second printer that was used in this study was the Eden 500V printer. This printer performs with the polyjet® method. The experimental models were printed with 16µm thick resin layers, which was the lowest layer thickness of this printer. In this type of printing technology, there is no final polymerization procedure for the model, as this occurs during the printing process. Ten models were printed from the Trios intraoral scanner, and ten models from Omnicam, totaling twenty models printed by the Eden 500V printer.

The plaster models were obtained from conventional impressions using polyvinyl siloxane (Express, 3M, Sumaré, Brazil) using the double impression technique. For that, a custom tray was made with a relief, in order to standardize the thickness of the impression material. Ten impressions were made, to obtain ten physical models of type IV plaster. Each impression generated only one plaster model. After molding, a type IV plaster (GC, Tokyo, Japan) was used. It was handled with the ratio of 20ml of water to 100g of plaster, what is recommended by the manufacturer. The plaster/water ratio was manipulated in a vacuum manipulator to avoid the inclusion of bubbles in the plaster and also to obtain a more homogeneous mixture. The plaster was poured into the mold and, after this process, 45 minutes were taken for the total crystallization, and it was then removed from the mold.

Four experimental groups were formed with ten models each (n = 10), according to the combination of the two printers and the two scanners. A fifth group, formed by the plaster models, was added. In view of the purpose of evaluating the quality of the models generated in the 5 different groups, the definitions trueness and precision expressed in the ISO-5725 (27) standard were used, to evaluate the capacity of the methods to generate precise and true models. The groups are shown in Table 1.

**Table 1:**
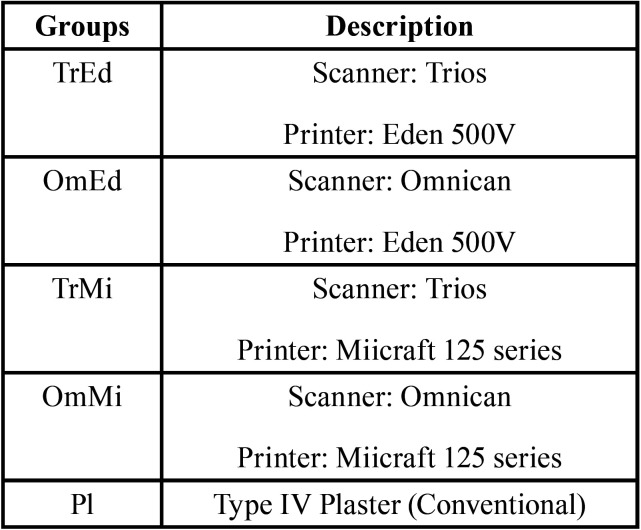
Description of the methods used to create the models.

**Figure 1:**
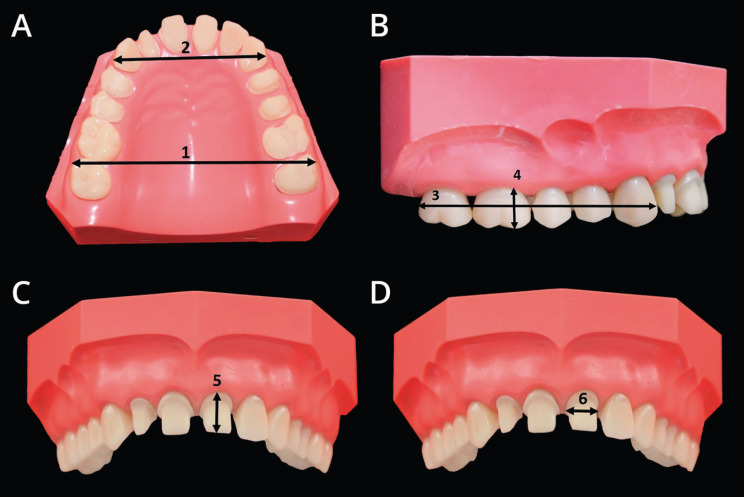
Dimension sites. A: dimension site 1 e 2; B: dimension sites 3 e 4; C: dimension site 5; D: dimension site 6.

Comparative measurements of the models of the five experimental groups (TrEd, OmEd, TrMi, OmMi and Pl) were performed using linear measurements at six dimension sites, located in different regions of the models (Fig. 1). These measurements were compared with the reference model, which served as reference.

Dimension site number 1 was the distance between the buccal surfaces of the Maxillary right second molar and the Maxillary left second molar. Dimension site number 2 was the distance between the distal part of the buccal surface of the Maxillary right canine and the Maxillary left canine. Dimension site number 3 was the distance between the distal surface of the Maxillary right second molar and the mesial surface of the Maxillary right canine. Dimension site number 4 was the height of the mesiobuccal cusp of the Maxillary right first molar. Dimension site number 5 was the cervical-incisal height of the preparation for the total crown of the Maxillary central left incisor and the dimension site 6 was the distance between the mesial and distal walls of preparation for total crown of the Maxillary central left incisor.

An image measuring machine called Quick Scope (Mitutoyo, Kawasaki, Japan) was used to perform measurements of physical models. This machine captures images and performs measurements based on the enlarged image of the physical model. Its camera system is polychromatic and determines the limits of the region to be measured by means of differences in image contrast, with an accuracy of 2.0 µm. It also features a fixed change magnification system by changing the type of lens, enlarging the obtained image without the use of an optical zoom, what improves the definition of the image. An individual adjustment of contrast and light was also performed. These steps were necessary due to the nature of the experimental models, composed of different types of materials, such as the Eden 500v printer resin, Miicraft125 series printer resin, the reference model resin and the type IV plaster of the Pl models.

In order to test the equivalence in the precision of the measurements made in the reference model and in the different models of the experimental groups, the Schuirmann Two-One Sided t test was applied in the standard deviation measurements. In the test, an upper equivalence limit of 0.1mm was established and the null value specified was that of the standard deviation of the three measurements made in the reference model.

In addition to the precision based on the idea of equality of the standard deviations, the trueness of the measurements of the experimental models was tested in comparison to those of the reference model by applying tests for paired data. The tests for applied paired data test the hypothesis that the difference between the means is equal to 0, proving that the measures of the reference model do not differ significantly from the experimental model under study. The test for paired data was selected by assessing the assumptions that underlie them, applying the Student’s t test on data adhering to the Gaussian distribution, the Wilcoxon test of the orders marked on data not adhering to the Gaussian distribution, but symmetrical, and the test of the signal when even the symmetry is not reasonable. In all statistical tests, the significance level of 5% (α = 0.05) was adopted and the calculations were made using the SAS system.

## Results

To evaluate the precision, the Schuirmann test was adopted with the hypothesis that the models have standard deviations similar to the standard deviations observed in the reference model. The results of the test are shown in Table 2.

**Table 2:**
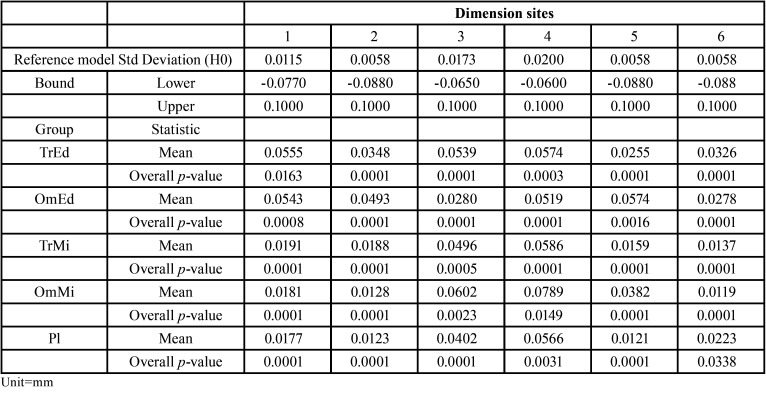
Standard deviations of the reference model, acceptable lower and upper limits for equivalent measures (Upper and Lower Bound) and, for each of the dimension sites, statistics of the standard deviations of the 5 groups (mean) and p-value for test of null hypothesis (Overall *p*-value).

The TrEd dimension site number 1, the OmMi dimension site number 4 and the Pl dimension site number 6, indications of equivalence (*p* <0.05) were observed between the standard deviations observed in the reference model. In all the others, there was a strong evidence (*p* <0.01) that the standard deviations observed in the groups are equivalent to the standard deviations of the measures taken directly in the reference model. Therefore, it can be established, within the adopted criteria, that the precision is similar in all methods.

After the precision assessment was completed, the trueness assessment was carried out. It was based on testing the existence of a difference between the measurements obtained in the experimental models in relation to the measurement obtained in the reference model. If there was a significant difference value of an experimental model dimension site in relation to the reference model (*p* <0.05), this difference could be positive, showing that the experimental model dimension site was larger than the reference model, or negative, showing that the experimental model dimension site was smaller than the reference model. The recommended tests were calculated and the results are shown in Table 3.

**Table 3:**
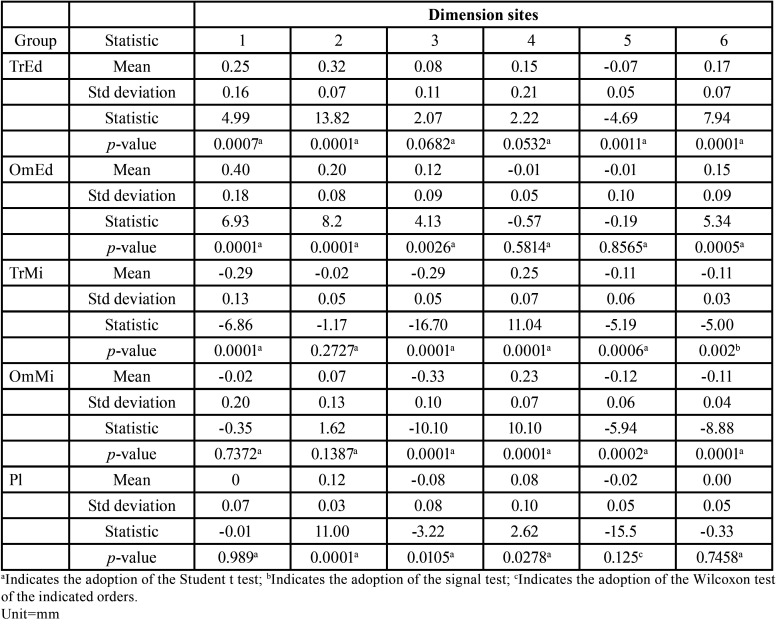
Mean, standard deviation (Std deviation), recommended statistic and p-value to test the null hypothesis of absence of differences in reference model measures to the experimental models measures (H0: µ0=0).

**Figure 2:**
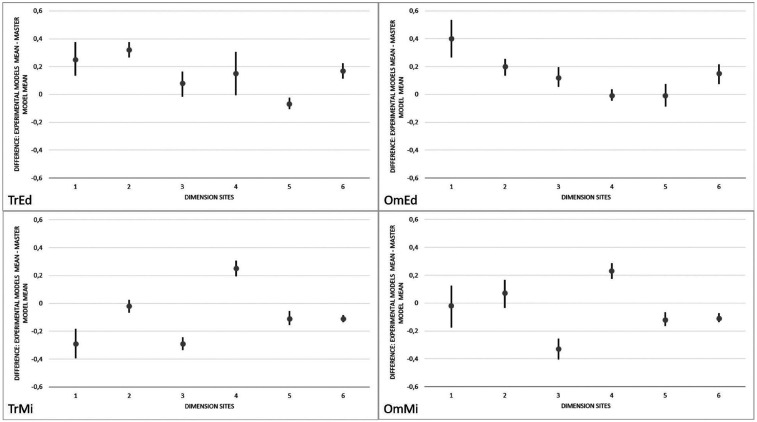
Average of the difference calculated between the measurements made in the reference model and in the TrEd, OmEd, TrMi and OmMi experimental models (confidence limits of the mean (95%) and tests for selected paired data).

Starting with TrEd, with the comparisons illustrated in Figure 2, it iwas observed that the averages of measures 1, 2 and 6 were significantly higher in the experimental models than in the reference model, and the average of measure 5 was significantly lower than that of the reference model. There were no signs of differences between measurements 3 and 4 of the reference model and the experimental models built by the TrEd method. The OmEd experimental models, there were signs of significant differences in measures 1, 2, 3 and 6. In all cases, the measurements obtained in the experimental models were greater than those observed in the reference model, with no evidence of differences in measures 4 and 5. In TrMi, there were signs that measures 1, 3, 5 and 6 were significantly lower in the experimental models than in the reference model and that measure 4 is significantly higher. There was no evidence of differences between the measurements at the dimension site number 2. The results of OmMi were very similar to those of TrMi, with the difference that measure 1 was not significantly different in this group. Moreover, there were signs that measures 3, 5 and 6 were significantly lower in the experimental models than in the reference model, when the test results were evaluated. Measure 4 was significantly higher in the experimental models than in the reference model. Also, there was no evidence of differences in measure 2.

**Figure 3:**
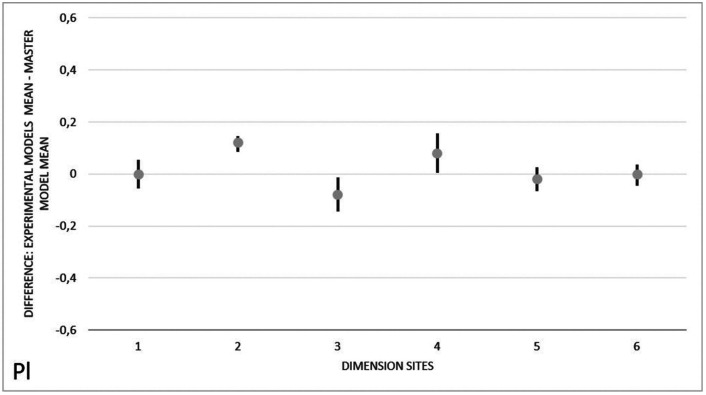
Average of the difference calculated between the measurements made in the reference model and in the Pl experimental models (confidence limits of the mean (95%) and tests for selected paired data).

The results of Pl (Fig. 3) showed the least amount of significantly different measures, since only measures 2, 3 and 4 showed significant diferences. Measures 2 and 4 were significantly greater in the reference model than in the experimental models while measure 3 was significantly lower. There was no evidence of differences between reference models and models in measures 1, 5 and 6.

## Discussion

The present study evaluated the accuracy of two methods of printed and type IV plaster models in comparison to a reference model. The methods generated models that presented similar measures to each other, within the same experimental group, in printed and plaster models. In relation to the printed models, the type of printer and the type of intraoral scanner did not negatively influence the production of physical models, with regard to precision.

There were problems with trueness in all methods of obtaining the experimental models. The results showed that both the printed models and the plaster models presented average measurements that were different from the reference model, within the same dimension site. There were sites that did not show significant differences with the reference model, but no experimental group showed absence of significant differences in all dimension sites. Therefore the null hypothesis was rejected, since the trueness of the printed and plaster models presented significant differences with the reference model.

Pl was the experimental group that presented the largest number of dimensions sites without significant differences with the reference model. This might mean that there is a tendency of PI presenting better accuracy, but not statistically significant. Studies found in literature indicate that plaster models show a better accuracy when compared to printed models (28,29).

A study found in the literature showed the influence of the layer height of the model in accuracy (30). The smallest layer height of models printed with Eden 500V was bigger than the smallest layer height of Miicraft 125 series, which led to a worse surface finish on Eden 500V. When compared to TrMi, OmMi and the plaster models, the printed models of TrEd and OmEd showed a worse surface finish. These results suggest that the type of printer might have influenced the results.

The type of scanner did not significantly influence the discrepancy values. TrEd and OmEd models were printed with Eden 500V printer (Polyjet method) and showed more sites with positive measurements (bigger than the reference model). TrMi and OmMi models, printed with the Miicraft 125 series printer (DLP method), presented more sites with negative measurements (smaller than the reference model), regardless of the type of scanner. Similar results were found in the literature (31). These outcomes might be related to the final polymerization process that the models printed by the Miicraft 125 series printer require. The process might have caused shrinkage of the models. The combination of printer and scanner must be analyzed to obtain a printed model with a great quality (32). However, the results suggest that the type of printer had a greater influence than the type of scanner in the accuracy of the printed models.

It should be pointed out that there were dimensions sites in the same experimental group in which significant differences occurred, and other sites within the same experimental group that did not present significant differences. Similar results were found in other studies (14). These results indicated that the dimension site also influenced the discrepancy of the models in this study. Dimension site number 1, which had a large dimension, was the site that presented the greatest dispersions in TrEd, OmEd and TrMi. In OmMi and Pl, this site did not show significant differences with the reference model, but it also showed great dispersion. Dimension sites with smaller dimensions, in general, had the smallest dispersions. Dimension site number 5 (small) did not present large dispersions in the average measures in any of the experimental groups.

This study was carried out with standardization of the entire process of obtaining printed and plaster physical models. The intra oral scanning process was performed by operators who were trained by the scanner manufacturers, reducing the influence of this factor on the quality of scanning (11). A single scanning strategy was also used, what reduced its influence on the quality of the digitized models (8,9). The printing process of the models was conducted in a standardized manner and in accordance with the specifications of the printer manufacturer.

The impression process was also carried out in a standardized manner and each mold generated only a single plaster model. The making of a custom tray was implemented in order to standardize the amount of impression material and generate more sTable molds, reducing the possibility of important dimensional changes. The double impression technique, used in this study, promoted the achievement of a mold with greater dimensional stability. All these factors when put together showed the importance of analyzing the process of obtaining the physical model as a whole, whether being printed or not.

For this is an *in vitro* study, it was not possible to simulate the clinical conditions that could impair the quality of the intra oral scanning process. Another limitation of the study is the fact that the superior performance of plaster models compared to printed models, regarding accuracy, may not be observed in the dentist’s routine. This is due to the fact that most dental surgeons do not perform the steps of manipulating and making the plaster model in a standardized way. There might be small errors in obtaining this model, which could lower the quality of it.

The influence of the process of obtaining the physical models, plaster or printed, is significant. Errors and dispersions are intrinsic to it and will always occur in the making of dental models. Another fact is that these inherent errors and variations in the making of a physical model should be at a clinically acceptable level. It is up to the dental surgeon to research which method will be the most suiTable for his/her purpose, knowing that these variations will occur.

## Conclusions

It can be concluded that the type of 3D printer was the factor that most influenced the accuracy of printed models. The type of intraoral scanner did not present significant influence. It was observed that the printed models showed similar precision to the plaster models, as well as trueness. Both techniques resulted in models with different measurements from the reference model, which impaired the accuracy of plaster and printed models.
